# Clinicopathological Spectrum of Ovarian Tumors and Diagnostic Utility of Cancer Antigen 125 (CA-125): An Observational Study From Eastern India

**DOI:** 10.7759/cureus.111404

**Published:** 2026-06-24

**Authors:** Akanksha Choudhary, Manoj K Paswan, Anshu Jamaiyar

**Affiliations:** 1 Pathology, Rajendra Institute of Medical Sciences, Ranchi, IND; 2 Ophthalmology, Rajendra Institute of Medical Sciences, Ranchi, IND

**Keywords:** ca-125 levels, diagnostic test accuracy, epithelial tumors, molecular biomarker, ovarian tumors

## Abstract

Background

Ovarian neoplasms represent a heterogeneous category of tumors with different histogenesis, clinical behavior, and prognosis, thus presenting a major challenge in gynecological oncology. The most popular biomarker in ovarian cancer is serum cancer antigen 125 (CA-125). Hence, the purpose of our study is to assess the diagnostic accuracy of serum CA-125 to differentiate between benign and malignant ovarian tumors using clinicopathological correlation in a tertiary care institution.

Methods

This is an observational study, retrospective in nature, carried out in the Department of Pathology at Rajendra Institute of Medical Sciences (RIMS), Ranchi, over 18 months. A total of 76 histopathologically confirmed ovarian tumor cases with available clinical, radiological, and serum CA-125 data were included. Preoperative serum CA-125 levels were measured using a cutoff value of 35 U/mL. Tumors were categorized as benign, borderline, or malignant based on histopathological criteria. Statistical analysis was performed using SPSS (IBM SPSS Statistics for Windows, IBM Corp., Version 27, Armonk, NY).

Results

Out of 76 ovarian tumors, 55 (72.4%) were benign, two (2.6%) were borderline, and 19 (25.0%) were malignant. Epithelial tumors (46, 60.5%) were the most common histological type. Serous cystadenoma (16, 21.1%) was the most frequent subtype. Elevated CA-125 levels were observed in all malignant tumors (19, 100%), while most benign tumors (49, 89.1%) showed normal levels. Serum CA-125 demonstrated sensitivity of 100%, specificity of 87.7%, positive predictive value of 73.1%, negative predictive value of 100%, and overall diagnostic accuracy of 90.7%.

Conclusion

The present study indicates that ovarian tumors are mainly benign, and most histological subtypes are epithelial tumors. Serum CA-125 demonstrated an excellent sensitivity in the detection of malignant tumors of the ovary, with all the malignant tumors portraying high levels of CA-125. These results show that CA-125 is a useful biomarker for identifying malignant ovarian tumors. However, its interpretation should be made in conjunction with clinical, radiological, and histopathological findings.

## Introduction

Ovarian neoplasms are a heterogeneous group of tumors whose histogenesis, clinical behavior, and prognosis are also diverse, making their management a great challenge in gynecological oncology. Ovarian cancer is one of the most common causes of cancer deaths in women due to the asymptomatic nature of the disease and late diagnosis at an advanced stage [1,2]. It is one of the most prevalent gynecological cancers in India, and is a significant cause of the cancer burden in the country [3]. The lack of specific clinical symptoms and the deep pelvic location of the ovary may cause a delay in diagnosis, which makes it worse for the survival outcome. Ovarian tumors actually originated from various cell elements of the ovary, namely the surface epithelium, germ cells, and sex cord-stromal elements [4]. As a result, these tumors demonstrate a wide histopathological spectrum ranging from benign cystic lesions to highly aggressive malignancies. Accurate preoperative differentiation between benign and malignant ovarian tumors is essential for appropriate clinical management and treatment planning. However, definitive diagnosis is primarily based on histopathological examination following surgical intervention.

Among the available tumor markers, serum cancer antigen 125 (CA-125) is the most widely used biomarker in ovarian cancer. CA-125 is a high-molecular-weight glycoprotein that is elevated in the majority of epithelial ovarian malignancies [5,6]. A serum cutoff value of 35 U/mL is commonly used in clinical practice. CA-125 has an established role in the diagnosis, monitoring, and prognostic assessment of ovarian tumors, particularly epithelial ovarian cancers [7,8]. Previous studies have demonstrated elevated CA-125 levels in nearly 80% of epithelial ovarian malignancies [7-9]. However, CA-125 has limitations, particularly in early-stage disease, premenopausal women, benign inflammatory conditions, and non-epithelial tumors. In addition, its sensitivity is relatively lower in early-stage disease. Variations in the reported diagnostic performance of CA-125 across studies may be attributed to differences in tumor biology, study populations, and methodological approaches [10,11].

However, CA-125 is a practical and affordable diagnostic tool, especially in low-resource areas where advanced imaging and molecular diagnostic tests may be unavailable. Clinicopathological correlation of ovarian tumors with CA-125 levels can be very useful in understanding the behavior of the tumors and to aid in accurate diagnosis. The clinicopathological characteristics of ovarian tumors and the role of CA-125 in their diagnosis are still not well-documented in these settings. The objective of our study was to estimate the clinicopathological profile of ovarian tumors and also to determine the diagnostic value of serum CA-125.

## Materials and methods

This observational study was carried out in the Department of Pathology, Rajendra Institute of Medical Sciences (RIMS), Ranchi, for 18 months from July 2024 to January 2026. This study included patients with clinically and radiologically suspected ovarian tumors that were subsequently confirmed by histopathological examination during the study period. Cases were selected by convenience sampling based on predefined eligibility criteria. Only patients with available clinical records, radiological findings, preoperative serum CA-125 levels, and adequate histopathological specimens were included in the analysis (n = 76). Patients were excluded if the tissue specimens were poorly fixed, inadequate for histopathological evaluation, or if essential clinical, radiological, or serum CA-125 data were unavailable. Clinical, radiological, laboratory, and histopathological information was retrieved from hospital records and pathology archives. The study was approved by the Institutional Ethics Committee (IEC) (IEC No. 112 dated 08/02/2024). As this was a retrospective record-based study utilizing anonymized patient data, individual informed consent was waived by the Institutional Ethics Committee.

Information regarding the clinical features, such as age, symptoms, parity, and menstrual history, was retrieved from the medical history of the hospital. Where available, the radiological data were recorded from ultrasonography and computed tomography reports. Serum CA-125 level was recorded before the operation in all patients, and a level of >35 U/mL was accepted as an elevated level. Standard pathological procedure (hematoxylin and eosin) was followed for the routine staining by a single pathologist blinded to CA125 values. Tumors were categorized as benign, borderline, or malignant and then subtyped based on the conventional histopathological criteria. Histopathological diagnosis of ovarian tumor was the primary outcome variable, and serum concentration of CA-125 was the main predictor variable. Other parameters studied were age, side of the tumor, clinical presentation, and histological subtype. Correlation of serum CA-125 levels with tumor behavior was performed to evaluate the association.

The data were entered into Microsoft Excel (Microsoft® Corp., Redmond, WA) and analyzed in SPSS (IBM SPSS Statistics for Windows, IBM Corp., Version 27, Armonk, NY). Data were presented in terms of descriptive statistics, including mean, standard deviation, frequencies, and percentages. Fisher’s exact test and chi-square test were used to perform inferential analysis. Receiver operating characteristic (ROC) curve analysis was performed using histopathological diagnosis of malignancy as the reference standard and preoperative serum CA-125 values as the test variable. Area under the curve (AUC) with 95% confidence intervals was calculated to assess discriminatory performance. A p-value of <0.05 was deemed statistically significant. The study was approved by the Institutional Ethics Committee (IEC) (IEC No. 112 dated 08/02/2024). All patient data were anonymized for analysis, and patient confidentiality was respected.

## Results

**Figure 1:**
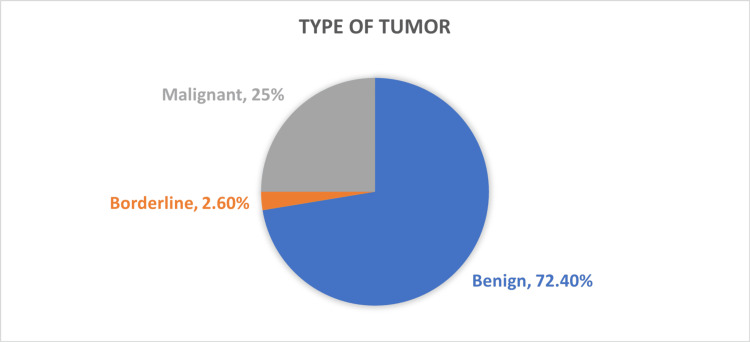
Distribution of ovarian tumors based on behavior

The study involved 76 ovarian tumor cases that were histopathologically diagnosed. The majority of cases (55, 72.4%) were benign, two (2.6%) were borderline, and 19 (25.0%) were malignant (Figure 1).

**Figure 2:**
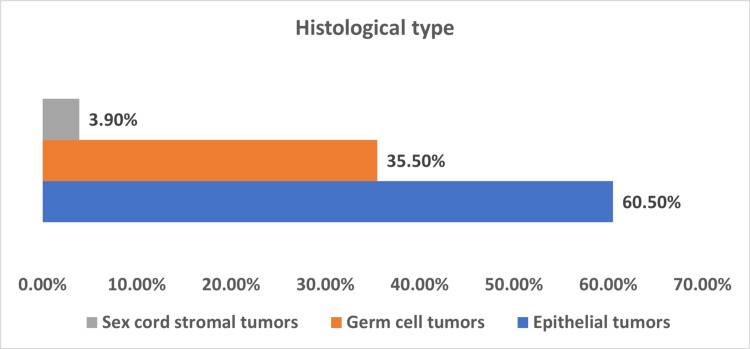
Distribution of ovarian tumors by histological category

Epithelial tumors were the most common histological category, observed in 46 (60.5%) cases, followed by germ cell tumors in 27 (35.5%) cases and sex cord-stromal tumors in three (4%) cases (Figure 2).

Among the histopathological subtypes, serous cystadenoma was the most common lesion, accounting for 16 (21.1%) cases, followed by mucinous cystadenoma in 14 (18.4%) cases and dermoid cyst in 10 (13.2%) cases. Among the malignant ovarian tumors, dysgerminoma was the most common histopathological subtype, accounting for five of 19 malignant cases (26.3%), followed by mucinous cystadenocarcinoma, papillary serous cystadenocarcinoma, yolk sac tumor, and adult granulosa cell tumor, each contributing two cases (10.5%).

Ovarian tumors were most frequently observed in the 31-40 years age group (23.6%), followed by the 21-30 years (19.7%) and 41-50 years (17.1%) age groups (Table 1).

**Table 1:** Age distribution of ovarian tumors

Age group	Number of cases	Percentage (%)
1-10 years	1	1.3
11-20 years	12	15.7
21-30 years	15	19.7
31-40 years	18	23.6
41-50 years	13	17.1
51-60 years	11	14.4
61-70 years	5	6.6
71-80 years	1	1.3

Abdominal pain was the most common presenting symptom, reported in 27 (35.5%) cases, followed by combined abdominal pain and lump in 25 (32.8%) cases. Six (7.9%) patients were asymptomatic (Table 2).

**Table 2:** Clinical presentation of ovarian tumors

Symptom	Number of cases	Percentage
Pain abdomen	27	35.5%
Lump abdomen	18	23.6%
Both	25	32.8%
No symptoms	6	7.9%

Ovarian tumors were more common among parous women (68.4%) compared with nulliparous women (31.5%).

Serum CA-125 levels were evaluated in relation to tumor behavior using a cutoff value of 35 U/mL. There were 50 cases with normal levels of CA-125, and 26 cases that were elevated. All malignant tumors were associated with elevated CA-125 levels, while 49 (89.1%) benign tumors had normal levels. The one borderline tumor had raised CA-125 (Table 3).

**Table 3:** Correlation of CA-125 levels with tumor behavior *Fisher’s exact test CA-125: cancer antigen 125

CA-125 level	Benign n (%)	Borderline n (%)	Malignant n (%)	Total n (%)	p-value*
<35 U/mL	49 (89.1)	1 (50.0)	0 (0.0)	50 (65.8)	<0.001
>35 U/mL	6 (10.9)	1 (50.0)	19 (100.0)	26 (34.2)	
Total	55 (100)	2 (100)	19 (100)	76 (100)	

Among the two borderline tumors, one showed an elevated CA-125 level (>35 U/mL), and one had a normal CA-125 level (<35 U/mL). Borderline tumors were included in the non-malignant category for diagnostic performance analysis. There were 50 cases with normal levels of CA-125, and 26 cases that were elevated. All malignant tumors were associated with elevated CA-125 levels, while 49 (89.1%) benign tumors had normal levels (Table 3). Analysis of tumor laterality showed that the right ovary was more commonly involved, accounting for 45 (59.2%) cases, followed by the left ovary in 29 (38.2%) cases. Bilateral involvement was observed in two (2.6%) cases. The diagnostic performance of serum CA-125 in differentiating malignant from non-malignant ovarian tumors is summarized in Table 4.

**Table 4:** Diagnostic performance of CA-125 in detecting malignant ovarian tumors CA-125: cancer antigen 125; NPV: negative predictive value; PPV: positive predictive value

Parameter	Value	95% CI
Sensitivity	100%	82.4%-100%
Specificity	87.7%	76.3%-94.9%
PPV	73.1%	52.2%-88.4%
NPV	100%	92.9%-100%
Accuracy	90.8%	81.9%-96.2%

Serum CA-125 demonstrated high sensitivity (100%) and negative predictive value (100%) for detecting malignant ovarian tumors. However, its lower specificity (87.7%) and moderate positive predictive value (73.07%) indicate the possibility of false-positive elevations in certain benign conditions.

**Figure 3:**
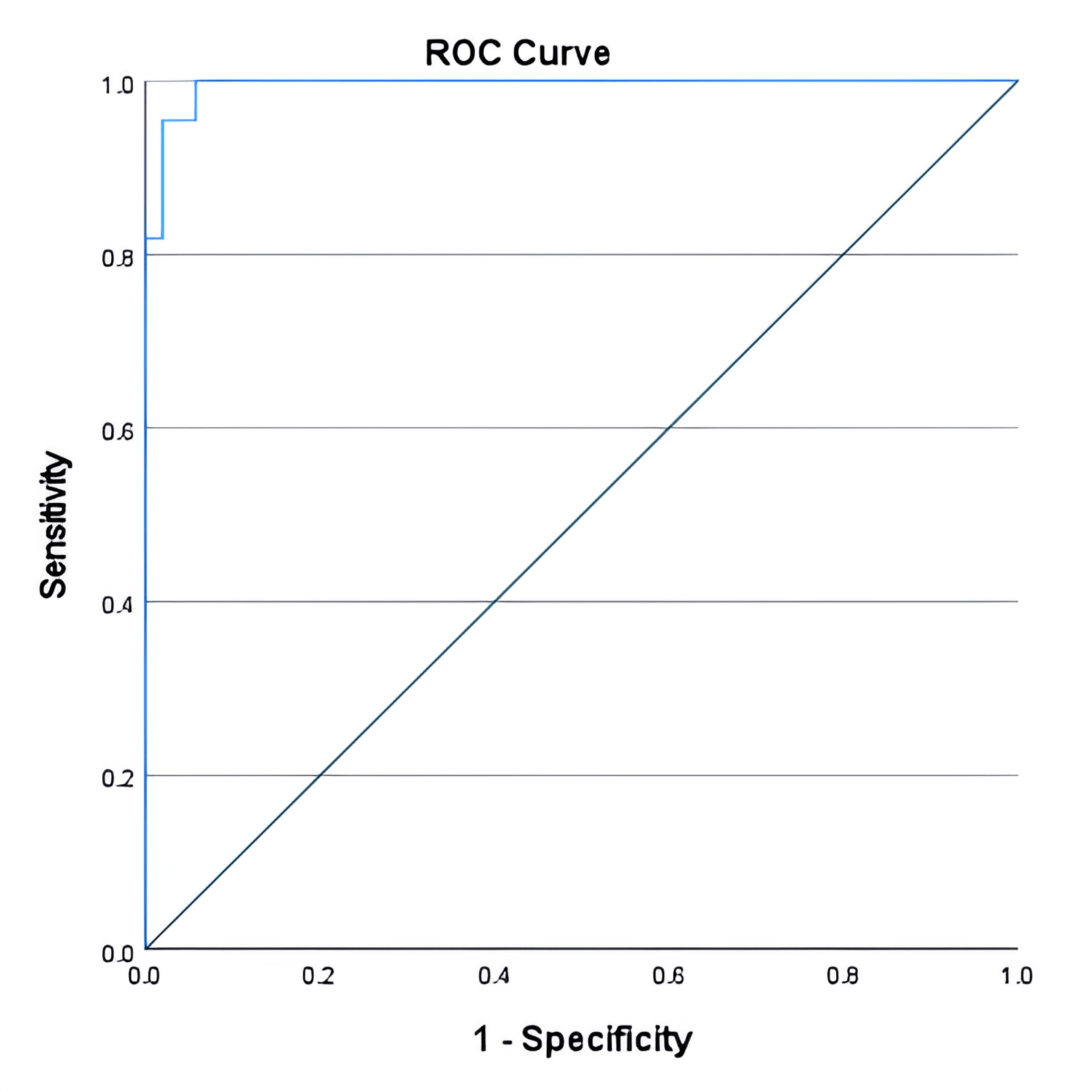
ROC curve demonstrating the diagnostic performance of serum CA-125 Area under the curve (AUC) of 0.995 (95% CI: 0.985-1.000; p < 0.001). A cutoff value of 35 U/mL was used for classification. CA-125: cancer antigen 125; ROC: receiver operating characteristic

ROC analysis showed excellent diagnostic accuracy of CA-125 with an AUC of 0.995 (95% CI: 0.985-1.000, p < 0.001), indicating near-perfect discrimination between malignant and non-malignant ovarian tumors (Figure 3).

## Discussion

Our study evaluated the clinicopathological spectrum of ovarian tumors and the diagnostic utility of serum CA-125 in a tertiary care setting. The predominance of benign tumors (55, 72.4%) over malignant tumors (19, 25.0%) in our study is comparable with the findings of Gupta et al. and Farag et al., who also reported a higher frequency of benign ovarian neoplasms [12,13]. This observation supports the established epidemiological pattern that most ovarian tumors are benign at the time of presentation. Epithelial tumors constituted the most common histological category (46, 60.5%), followed by germ cell tumors (27, 35.5%) and sex cord-stromal tumors (three, 3.9%). Similar findings were reported by Gupta et al., who observed epithelial tumors as the predominant subtype in 71.7% of cases [12]. However, the relatively higher proportion of germ cell tumors in our study may be attributed to the younger age distribution of the study population. Among the histopathological subtypes, serous cystadenoma (16, 21.1%) was the most common lesion, followed by mucinous cystadenoma (14, 18.4%) and dermoid cyst (10, 13.2%). These findings are consistent with previous studies by Gupta et al., which reported a similar distribution of ovarian tumor subtypes [12]. The highest incidence of ovarian tumors was observed in the 31-40 years age group (18, 23.6%), which is in agreement with the studies by Farag et al. and Chanu et al., who also reported increased prevalence during the reproductive age group [13,14]. Abdominal pain was the most common presenting symptom (27, 35.5%), comparable to the observations of Rao et al. [15], whereas Mannan et al. reported abdominal mass as the predominant clinical presentation [16], suggesting variability in clinical manifestations across different populations.

A major finding of our study was the significant association between elevated serum CA-125 levels and malignant ovarian tumors, with all malignant cases (19, 100.0%) demonstrating CA-125 levels >35 U/mL. This finding is higher than that reported by Prakash et al., who observed CA-125 positivity in 65.4% of malignant tumors [10], but is comparable with the observations of Funston et al., who reported elevated CA-125 levels in nearly 80% of epithelial ovarian cancers [8]. Furthermore, significantly higher serum CA-125 levels in malignant tumors compared with benign lesions, as reported by Patil et al., further support its diagnostic utility [17]. Despite its high sensitivity, CA-125 demonstrated limited specificity, as six (10.9%) benign tumors also showed elevated levels. Similar findings were reported by Sjövall et al., who documented elevated CA-125 levels in both malignant and benign ovarian conditions [9]. This highlights the limitation of CA-125 as a standalone diagnostic marker. Elevated CA-125 levels observed in certain malignant germ cell and sex cord-stromal tumors in our study suggest that the marker may reflect tumor burden rather than tumor subtype specificity. Right-sided ovarian involvement (45, 59.2%) was more common in our study, which is comparable to the findings of Pilli et al. [18], although the exact reason for this laterality pattern remains uncertain.

Our study has several strengths: all cases were histopathologically confirmed, a thorough clinicopathological correlation was attempted, and several histological types of ovarian tumors were included. The observed high sensitivity of our study may be attributed to the advanced presentation stage or to the predominance of epithelial tumors. Some of these constraints should be acknowledged, such as the relatively small sample size, observational single-center design, and absence of tumor staging and further biomarker analysis, which may reduce the generalizability of the findings, and the International Federation of Gynecology and Obstetrics (FIGO) stage for malignant tumors was unavailable for the cases. The use of convenience sampling and inclusion of only cases with complete clinical, radiological, serum CA-125, and histopathological data may have introduced selection bias. As this was a retrospective study, detailed information regarding the assay platform, manufacturer specifications, and quality-control procedures used for CA-125 estimation was not available from the archived laboratory records. This limitation may affect the reproducibility of the findings across different laboratory settings. None of this staging data is available either, which limits the interpretation of CA-125 performance in early-stage versus advanced-stage disease. The exceptionally high AUC observed in the present study should be interpreted cautiously because of the relatively small sample size and single-center design. External validation in larger cohorts is required before generalizing this diagnostic performance.

## Conclusions

Our study reveals that most of the ovarian tumors are benign, and that the major histological type of the tumors is epithelial. In the detection of malignant ovarian tumors, serum CA-125 was highly sensitive, and all the malignant cases exhibited elevated levels of CA-125. False positive elevations were seen in a small percentage of non-cancerous conditions; however, its specificity was relatively low. These data conclude that CA-125 had excellent sensitivity and moderate specificity in differentiating malignant ovarian tumors and is a useful adjunct in combination with clinical, radiological, and histopathological findings.
